# Clinical Neuropathology image 5-2016: neurofibrillary tangle-rich ganglioglioma 

**DOI:** 10.5414/NP300982

**Published:** 2016-08-22

**Authors:** Ellen Gelpi, Romana Höftberger, Tanja Würger, Johannes Kerschbaumer, Christian F. Freyschlag, Tanja Djurdjevic, Johannes A. Hainfellner

**Affiliations:** 1 Institute of Neurology, Medical University of Vienna, Vienna, Austria,; 2 Neurological Tissue Bank of the IDIBAPS Biobank, Barcelona, Spain,; 3 Institute of Pathology, Medical University of Vienna, Vienna,; 4 Department of Neurosurgery, and; 5 Department of Neuroradiology, Medical University of Innsbruck, Innsbruck, Austria; *Both authors contributed equally

**Keywords:** ganglioglioma, neurofibrillary tangle, hyperphosphorylated Tau

## Abstract

No Abstract available.

We present radiological and neuropathological features of an incidental finding in a 64-year-old woman. She underwent brain MRI due to a short-lasting episode of dizziness and nausea. A small diffuse left frontal lesion, hyperintense in T2 with focal well-delineated contrast enhancing area was detected (Figure 1). Histology showed a cortico-subcortical tumor nodule with two cellular components: middle-sized, elongated cells with fine fibrillar, bipolar processes intermingled with clusters of large, dysmorphic/dysplastic ganglion cells, both embedded in a fibrillary matrix (Figure 2A). Dystrophic calcifications were also frequently observed as well as focal perivascular lymphocytic cuffing. Many of the neuronal cells harboured large basophilic and fibrillar inclusions in their cytoplasm, consistent with neurofibrillary tangles (NFTs) (Figure 2B, C). These were strongly immunoreactive for p62 and for hyperphosphorylated τ (AT8) (Figure 2D). This staining also showed abundant τ-immunoreactive cell processes/threads. NFTs were partly positive for phosphorylated neurofilaments (SMI31) (Figure 2E) and synaptophysin (Figure 2E). No abnormal α-synuclein, TDP43, PrP, or β-amyloid deposits were detected. CD34 did not stain the neuronal elements but labelled focally the fibrillary matrix. The MIB-1 proliferation index was 1%. The final diagnosis was ganglioglioma WHO grade I with abundant neurofibrillary tangles. 

Neuronal neurofibrillary tangles are usually detected in a subgroup of primary neurodegenerative diseases [1] (e.g., Alzheimer’s disease, progressive supranuclear palsy, corticobasal degeneration, primary age-related tauopathy) but also in other, etiologically-diverse conditions such as chronic traumatic brain injury, Fahr’s disease, myotonic dystrophy subacute sclerosing panencephalitis [1], or slowly growing focal brain lesions such as meningoangiomatosis [2] or low grade gangliogliomas [3], as shown here. It is postulated that a chronic insult to neurons may trigger hyperphosphorylation of the microtubule associated protein τ, inducing the assembly of filaments and their accumulation in form of fibrils. This may lead to destabilization of the neuronal cytoskeleton, to neuronal dysfunction, and finally to neuronal death. 

**Figure 1.**
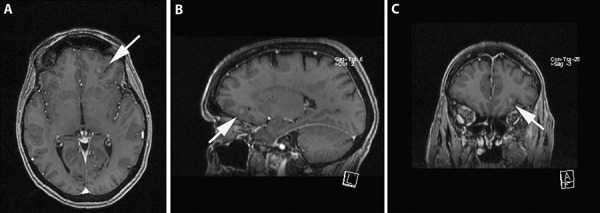
MRI showing a diffuse lesion left frontal on T1 sequences with focal contrast enhancement (A: axial, B: sagittal, C: coronal view).

**Figure 2.**
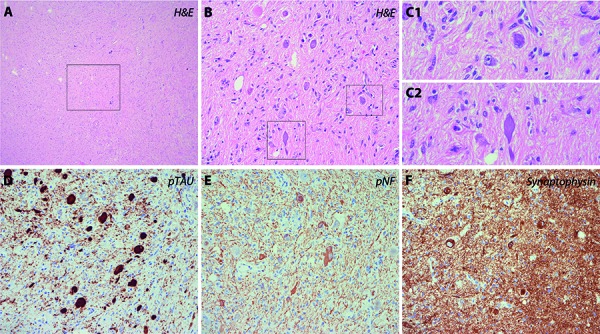
Histological images of the nodular lesion with formation of abundant neurofibrillary tangles.
